# Editorial: Climate change challenge: adaptation to climate change

**DOI:** 10.3389/fpsyg.2025.1675673

**Published:** 2025-09-19

**Authors:** Giuseppe Carrus, Chiara Massullo, Lorenza Tiberio, Lina Fusaro, Christoph Steinebach

**Affiliations:** ^1^Department of Education, Roma Tre University, Rome, Italy; ^2^Institute of BioEconomy (IBE), National Research Council, Rome, Italy; ^3^Zürich University of Applied Sciences, Zurich, Switzerland

**Keywords:** climate change, adaptation, resilience to climate change, ecological behavior, wellbeing, sustainability

Climate change is one of the defining challenges of our time and requires a multidisciplinary approach to understand and address it. Psychology plays a central role in understanding human behavior in relation to the transition to lower-carbon lifestyles and adaptation to the effects of climate change. This Research Topic has brought together recent empirical studies highlighting the current state of research on climate change adaptation, from various psychological perspectives, including environmental psychology, social psychology, clinical psychology, and developmental psychology, incorporating findings from other sciences, and following up on previous similar collections published in this section (e.g., Pirchio et al., 2020) or other sources (e.g., Carrus et al., 2020; De Gregorio et al., 2023). However, the focus here is on the psychological, educational, and design implications of adaptation strategies across various cultures and regions of the world. In addition, the papers included in the present Research Topic suggest how psychological insights can influence interventions to strengthen resilience and promote collective action, at various levels, such as local and global (from the environmental and economic point of view), or individual, collective and societal (from the psychological point of view). Likewise, the variety of empirical and theoretical contributions of this Research Topic describes the different possible types of mechanisms supporting climate change action, such as preventive, responsive, or recovery resilience functions, for example.

At a global level, an interesting question we have addressed in this Research Topic is the following: what contribution can psychology make to achieving the UN Sustainable Development Goals (SDGs)? Psychologically, this involves questions related to the perception of the different aspects of climate change, and of the related implications in terms of developing adequate programs of education and training for ecologically appropriate behavior, as well as setting up the conditions for increased collective responsibility and implementing effective interventions to promote environmentally conscious attitudes and behaviors. This aspect also touches the problem of environmental inaction and attitude-behavior gaps in pro-environmental action (e.g., Klöckner, 2013) which, in turn, has been linked to the issue of climate change skepticism and anti-scientific stances (e.g., Gligorić et al., 2025). According to various studies, climate change denialism may also be conflated with political and ideological orientations (e.g., Carrus et al., 2018a), especially in the USA, while apparently less so in other parts of the world (e.g., Calonge-Reillo, 2025; Hornsey et al., 2018).

At a more local level, it is also important to understand how specific psychological concepts can be applied to create conditions for a more sustainable adaptation to climate change processes, in real life settings. From this point of view, resilience has proven to be a successful adaptation strategy, functioning at different levels, and allowing for different adaptation strategies in the relation between the individuals and their surrounding environments (e.g., Steinebach and Langer, 2019).

Human resilience in relation to climate change may in fact operate at multiple levels, each contributing to enhance our adaptive capacity to cope with increasingly demanding environmental conditions. At an individual level, resilience may imply a personal readiness to undertake adaptive behaviors in different domains, such as more sustainable consumption or more sustainable technology adoption, although negative trade-offs between climate change adaptation and mitigation measures may also occur (e.g., Moser, 2012). At a collective level, resilience processes have been related to social networks, place attachment and identity, community ties and local initiatives, which may have a positive role for the promotion of adaptive capacity and collective efficacy in relation to environmental challenges. Specific features of the physical environment can also help resilience in relation to climate change, such as the presence of nature in urban settings (Hartig et al., 2014), walkability (Brown et al., 2007) or other urban affordances (e.g., Carrus et al., 2018b). These different features of resilience can in turn help to the develop preventive, responsive or recovery strategies, across different types of climate impacts, and focusing either on the reduction of risks, the addressing of primary needs, and the recovery of functional resources.

Psychologically, resilience is defined as the positive coping of a system in its environment in the sense of a sustainable change, in order to respond appropriately to short-term or long-term everyday challenges or severe stresses. Based on internal processes, the system interacts with the environment to define new reference values, develop the necessary competencies for self-development and positive adjustment to environmental conditions, and improve its ability to cope well with future challenges. In the context of climate change, such resilient adaptation also includes processes of accommodation and assimilation. We must not forget that, in addition to climate change, other risks also require special attention in present-day globalized human societies. If we follow the risk analyses of the World Economic Forum, for example, there are short- and medium-term risks associated with the cost of living, natural disasters and extreme weather events, the failure of climate protection measures, the erosion of social cohesion and societal polarization, the consequences of large-scale involuntary migration, widespread cybercrime and cyber insecurity, as well as the depletion of natural resources and large-scale environmental damage. All these risks may emerge independently of each other, but they also reinforce each other, thereby amplifying their negative effects.

Against this backdrop, the articles in this Research Topic address some very fundamental questions: what role do environmental emotions play when it comes to mental health or environmental activism? These articles examine the concept of ecological emotions, i.e., emotions that people experience in response to the environmental crisis (see articles listed in Table 1 as n. 2, 7, 9, 10, 12). It is argued that these emotions, such as eco-anxiety and eco-grief, are natural reactions and can serve as motivators for collective action. Eco-generativity is a concept that examines the negative emotional response to climate change (eco-anxiety). The authors propose eco-generativity as a way of dealing with eco-anxiety by focusing on finding solutions and working toward a sustainable future. Climate change anxiety (CCA) is a specific type of anxiety caused by the threat of climate change. While messages that focus on the negative consequences of climate change can increase anxiety, anxiety alone does not necessarily lead to action. Anger is seen as a more important factor in motivating people to act against climate change. A new tool for measuring climate change skepticism has been developed that captures beliefs related to climate change denial. The study found that political ideology is the most consistent predictor of climate change skepticism, and that “dark” personality traits also correlate with climate change skepticism.

**Table 1:** List of articles included in this RT.

**1) Albrecht, S. L., Donnelly, T., Frenkiel, M., Rajic, S. K., Kavadas, V., and Leiter, M.P. (2023). Pro-environmental employee engagement: the influence of pro-environmental psychological capital, pro-environmental job resources, and perceived corporate environmental responsibility. *Front. Sustain*. 4:1117892. doi:10.3389/frsus.2023.1117892**
2) Borzino, N., Chng, S., and Schubert, R. (2025). Outdoor thermal comfort and cognition impact pro-environmental behaviors: evidence from a field experiment in the tropics. *Front. Psychol*. 16:1472852. doi:10.3389/fpsyg.2025.1472852
3) Branham, L. (2024). Embodied earth kinship: interoceptive awareness and relational attachment personal factors predict nature connectedness in a structural model of nature connection. *Front. Psychol*. 15:1400655. doi:10.3389/fpsyg.2024.1400655
4) Cunha, J., Martins, J., Núñez, J. C., Vallejo, G., and Rosário, P. (2025). Adolescents' agency toward climate change: development and validation of scales for individual, proxy, and collective modes. *Front. Psychol*. 16:1532409. doi:10.3389/fpsyg.2025.1532409
5) Han, J., Sun, R., Zeeshan, M., Rehman, A., and Ullah, I. (2023). The impact of digital transformation on green total factor productivity of heavily polluting enterprises. *Front. Psychol*. 14:1265391. doi:10.3389/fpsyg.2023.1265391
6) Harcourt, R., Dessai, S., Bruine de Bruin, W., and Taylor, A. (2023). A social science research agenda to accelerate public engagement in climate change adaptation. *Front. Psychol*. 14:1286525. doi:10.3389/fpsyg.2023.1286525
7) Di Fabio, A., and Svicher, A. (2024). The challenge of eco-generativity. Embracing a positive mindset beyond eco-anxiety: a research agenda. *Front. Psychol*. 15:1173303. doi:10.3389/fpsyg.2024.1173303
8) Leka, J., and Furnham, A. (2024). Correlates of climate change skepticism. *Front. Psychol*. 15:1328307. doi:10.3389/fpsyg.2024.1328307
9) Lerolle, A., Micoulaud-Franchi, J.-A., Fourneret, P., Heeren, A., and Gauld, C. (2025). Exploring the relationship between eco-anxiety and suicide risk in adolescents with mental health disorders: insights from a cross-sectional observational study. *Front. Psychol*. 15:1408835. doi:10.3389/fpsyg.2024.1408835
10) Lundheim, S. H., and Löfström, E. (2025). Wind energy development in Norway: exploring the emotional landscape. *Front. Psychol*. 16:1386921. doi:10.3389/fpsyg.2025.1386921
11) Muffato, V., Miola, L., Pazzaglia, F. and Meneghetti, C. (2025). Are explorers greener? Investigating the role of personality traits, connectedness to nature and attitudes toward exploring in various pro-environmental behaviors. *Front. Psychol*. 15:1404095. doi:10.3389/fpsyg.2024.1404095
12) Qiu, S., and Qiu, J. (2024). From individual resilience to collective response: reframing ecological emotions as catalysts for holistic environmental engagement. *Front. Psychol*. 15:1363418. doi:10.3389/fpsyg.2024.1363418
13) Serpa-Barrientos, A., Pérez-Flores, E. G., Bellido-Figueroa, G. M., and Saintila, J. (2023) Evidence of validity and reliability of the environmental action scale in Peruvian university students. *Front. Psychol*. 14:1232397. doi:10.3389/fpsyg.2023.1232397
14) Simon, C. E., and Merten, M. J. (2024). Better climate action through the right knowledge? Development and validation of an item-response-theory scale measuring climate effectiveness knowledge. *Front. Psychol*. 15:1347407.doi:10.3389/fpsyg.2024.1347407
15) Thomson, E. E., and Roach, S. P. (2023). The relationships among nature connectedness, climate anxiety, climate action, climate knowledge, and mental health. *Front. Psychol*. 14:1241400. doi:10.3389/fpsyg.2023.1241400
16) Tian, J., Zheng, X., and Sun, Y. (2023). Fostering public climate change discussions from a social interaction perspective. *Front. Psychol*. 14:1258150. doi:10.3389/fpsyg.2023.1258150
17) von Gal, A., Fabiani, G., and Piccardi, L. (2024). Climate change anxiety, fear, and intention to act. *Front. Psychol*. 15:1341921. doi:10.3389/fpsyg.2024.1341921
18) Weihgold, V. (2024). Moral submissiveness: social origin as a vulnerability for well-being on a warming planet. *Front. Psychol*. 15:1355736. doi:10.3389/fpsyg.2024.1355736
19) Miller, L. B., and Rice, R. E. (2025). Psychological distance and pro-environmental behavior: insights from wildfire-affected PCT hikers. *Front. Psychol*. 16:1481964. doi:10.3389/fpsyg.2025.1481964

Another important theme addressed in this Research Topic related to the role played by social discourse and social networks, and how can a meaningful public discussion about climate change be promoted (see articles listed in Table 1 as n. 1, 4, 6, 8, 16, 18). Positive social interaction is in fact crucial for promoting healthy public discussions on climate change. People are more likely to talk about climate change if they believe their conversations will be effective. Social norms also play a role, as people are more likely to participate in climate change discussions if this is common in their social group. The articles in this Research Topic also examine the relationship between moral submissiveness, social origin, and wellbeing in the context of climate change. These studies show that people from less advantaged social groups are more likely to develop a sense of learned helplessness, which can make them more vulnerable to the negative effects of climate change. Moral submissiveness is the tendency to submit to others even when it goes against one's own morals, and can lead to learned helplessness, where one believes to have no control over negative outcomes in life. To promote wellbeing in the context of climate change, it is therefore crucial to consider specific social factors that influence people's wellbeing, such as moral attitudes and sense of agency.

But how do these factors change environmental behavior? This question is addressed by articles listed in Table 1 as n. 3, 5, 11, 13, 14, 15, 17, 19.

In high-income countries, more and more people view climate change as a personal threat. However, there is still a gap between awareness and action. Psychological distance may be crucial in this regard, as showed in this Research Topic: the higher the psychological distance, the lower the likelihood of developing climate-related attitudes and undertaking appropriate climate behaviors. Articles published in this Research Topic also propose a research agenda to understand what motivates people to take action against climate change. Research on the relationships between nature connection, climate anxiety, and climate action also shows that climate anxiety can be associated with poor mental health, but that nature connection can influence climate action. Climate competence also plays a role, as nature connection seems to work positively especially for people with adequate climate knowledge. To this aim, the cross-cultural validation of standardized measurement tools is also important, such as in the case of the Environmental Action Scale (EAS), which measures an individual's self-reported commitment to environmental behavior. As suggested by a study applying the EAS to Peruvian university students, this scale may be a reliable and valid instrument for measuring environmental actions in different populations and cultural and geographical contexts.

From a more organizational point of view, researches collected here also examine the factors that influence employee engagement in corporate environmental measures and how societal and macroeconomic scenarios (such as example digitalization) may interact with individual behaviors in relation to climate change (see articles listed in Table 1 as n. 1 and 5). For example, a model is proposed that integrates perceived corporate environmental responsibility, environmental work resources, and environmental psychological capital to predict employees' environmental commitment. The results of this study show that all three factors positively influence employees' environmental commitment. Digital transformation may also contribute to climate change adaptation, as research in this Research Topic seem to suggest. In highly polluting companies, digital transformation can for example improve the green total factor productivity (GTFP, which is a measure of a company's environmental and economic efficiency). Digital transformation can significantly improve GTFP by promoting green innovation and management efficiency, thus reducing external transaction costs. However, the effects of digital transformation are heterogeneous across industries.

In conclusion, we believe that the articles included in this Research Topic, covering a wide range of conceptual approaches, methods and disciplinary backgrounds, represent a good starting point to set up the discussion about the psychological factors related to human adaptation to climate change, and an interesting benchmark fort future empirical studies and theoretical reflections about the psychological antecedents and consequences of climate change adaptation.
